# Cognitive CAMSA: an ecological proposal to integrate cognitive performance into motor competence assessment

**DOI:** 10.3389/fpsyg.2023.1330856

**Published:** 2023-12-22

**Authors:** Sergio Montalt-García, Isaac Estevan, Jorge Romero-Martínez, Nuria Ortega-Benavent, Israel Villarrasa-Sapiña, Cristina Menescardi, Xavier García-Massó

**Affiliations:** ^1^AFIPS Research Group, Department de Didàctica de l’Educació Física, Artística i Música, Universitat de València, València, Spain; ^2^HUMAG Research Group, Department d’Educació Física i Esportiva, Universitat de València, València, Spain; ^3^HUMAG Research Group, Department de Didàctica de l’Educació Física, Artística i Música, Universitat de València, València, Spain

**Keywords:** motor competence, cognitive performance, physical literacy, dual-task, adolescent profiles

## Abstract

**Purpose:**

To profile the participants using a system of self-organizing maps (SOM) based on their motor and cognitive performance during a dual-task version of the Canadian Agility and Movement Skill Assessment (Cognitive CAMSA).

**Methods:**

A total of 169 secondary school students (39.3% girls) volunteered to participate. The original CAMSA, cognitive CAMSA, the Corsi and Digit Span tests were used to assess (a) motor competence, (b) motor competence with cognitive load, and (c) cognitive performance, respectively. SOMs and the *k*-means clustering algorithm were used to establish the adolescents’ dual-task performance profiles.

**Results:**

Including decision making based on verbal and visual cues in the original CAMSA significantly increased the participants’ total scores but also the time required to complete the test, while the skill score remained unchanged. However, not all the participants showed changes in their performance in the same direction during the cognitive CAMSA. Person-centered analyses by SOMs and *k*-means clustering identified six performance profiles with variations in the cognitive, motor skill, and time scores (H_5_ = 146.15, H_5_ = 102.68, and H_5_ = 108.07, respectively; all *p* < 0.01).

**Conclusion:**

The cognitive CAMSA was shown to be a feasible field-motor test for assessing motor competence with a cognitive load in an ecological setting. Some of the profiles identified in the SOM approach represented adolescents with similar motor and cognitive performance in dual-task or single-task contexts, although other participants obtained high motor competence in single and dual-tasking while their cognitive performance declined or rose more in dual-task than in single task situations. The cognitive CAMSA emerges as a tool of great potential, applicable in educational and sports environments, to know subjects’ characteristics and try to individualize the interventions accordingly with their dual-task profile.

## 1 Introduction

The concept of Physical Literacy (PL) can be defined as the set of social, emotional and cognitive capacities to cooperate and communicate appropriately with the environment. It also requires a holistic commitment including integrated physical capacities in perception, experience, memory, anticipation, and decision making (Whitehead, 2001). PL currently refers to motivation, confidence, physical competence, knowledge, and understanding to value and take responsibility for engagement in physical activities for life (Cairney et al., 2019; Whitehead, 2019). PL also includes four interconnected domains, i.e., psychological, social, cognitive, and physical (Longmuir et al., 2015). Achieving appropriate levels of PL is a fundamental part of developing self-fulfillment, self-confidence, and positive self-esteem.

Physical education pays particular attention to motor competence, which is a key element in the PL domain. Although there is a wide variety of tests available to evaluate motor competence, they do have some limitations (Hulteen et al., 2020). Whereas isolated-based field-motor tests (e.g., the Gross Motor Development Test or the Körperkoordinations Für Kinder Test) are reliable enough in assessing motor competence, others are too far removed from the context in which fundamental movement skills are carried out (usually situations in sport that require a combination of skills). Circuit-based field-motor tests emerged as an alternative system of evaluating children’s movement skills and their ability to combine simple movements to perform more complex movements [e.g., the Canadian Agility and Movement Skill Assessment (CAMSA); Longmuir et al., 2017]. However, as making decisions, which is inherent in practicing sport, is missing in the CAMSA it could be argued that this test do not measure motor competence in a completely ecological manner. Including perception tasks and decision making in the original version of the CAMSA test would be an interesting way of evaluating motor competence in an ecological setting as well as evaluating some of the PL cognitive elements (e.g., perceptual awareness or reasoning; Barnett et al., 2020).

To date, no test has been devised to concisely assess the cognitive domain. In fact, Cornish et al. (2020) states that the instruments used to measure PL usually focus on fundamental movement skills and are limited in measuring the cognitive domain (Robinson and Randall, 2017). It is also suggested that future research should attempt to explore and develop instruments to assess the cognitive domain objectively to understand motor development and PL holistically (Edwards et al., 2018). Cognitive tests are usually carried out to assess children’s executive functions, such as working memory, inhibition, and cognitive flexibility (Diamond, 2013). However, these tests are performed in a sedentary context far removed from physical activity. As the results of these tests may possibly differ from the cognitive performance during physical activity, we therefore considered it would be of interest to develop an instrument capable of evaluating elements in the cognitive domain when carrying out motor competence tests.

Combining cognitive and physical tasks in the same test seems to be the key to creating a method of ecologically determining PL cognitive level and performance, while dual-tasking (DT) is an already existing paradigm in which motor and cognitive tasks are carried out simultaneously and the effect of the interactions between them on performance are studied (Bustillo-Casero et al., 2017). DT is used in laboratory settings but its usefulness in more ecological environments is still unknown. Tests that include simultaneous motor and cognitive tasks could be used to assess cognitive function and motor competence ecologically. It is also of interest to design tools that allow PL to be assessed holistically, or at least gradually incorporate multiple domains simultaneously. Using DT to assess both motor and cognitive performance in the same field test would be a breakthrough in achieving this goal.

Even though it is important to consider that the interference generated by performing two tasks at the same time may affect the students who are proficient in performing simple tasks and who may not be as proficient in a DT, different interaction patterns have been found between tasks during DT. In these models, we find three different theories to explain DT interference (Lacour et al., 2008; Bonnet and Baudry, 2016; Wollesen et al., 2016). These include the cross-domain competition model (two tasks performed simultaneously or in quick succession, requiring the same or overlapping cognitive processes), the inverted U-shaped nonlinear interaction model (the optimal level of task difficulty that allows efficient multitasking, while both easier and more difficult tasks can result in reduced performance), and the prioritization model (faced with multiple tasks, individuals allocate most attention to the most important at a given time while reducing the resources allocated to the lower-priority task). These three models emerged as the outcomes of divergent results in different DT studies (Lacour et al., 2008; Bonnet and Baudry, 2016; Wollesen et al., 2016; Bustillo-Casero et al., 2020). The divergence in the findings could have been the result of the subjects’ individual strategies to deal with the DT. In fact, we do not know how factors like age or task difficulty can influence performance during DT (Schaefer, 2014; Bustillo-Casero et al., 2017; Marco Ahulló et al., 2022).

The aim of this study was thus to design and test a new version of the CAMSA that included decision-based verbal and visual cues (i.e., cognitive CAMSA) to assess both motor competence and cognitive performance, plus the impact of the cognitive load in motor competence in an ecological setting. To achieve this, the interference in the students’ performance in the variables derived from the original CAMSA (i.e., motor skill score, time score, and total score) when performing the cognitive version of the test was first determined. It was thus possible to observe whether the students responded differently when performing the traditional test compared to the cognitive CAMSA in the motor domain. On confirming that not all the students responded in the same way to a DT, the second objective was to establish the student profiles based on the variables derived from the cognitive CAMSA (i.e., cognitive score, motor skill score, and time spent). The third objective was to determine the differences between the performance profiles in single cognitive tasks, single motor tasks, performance during DT, and gender, which would allow us to determine whether the PL cognitive and motor tests using DT aligned with the results of single tests. If we confirmed that different results were obtained in DT and single tasks, it would open up a discussion about the suitability of DT-based tests to assess cognitive and motor performance. DT could thus provide a more ecological assessment and would better represent what actually happens during physical activities and sports.

## 2 Materials and methods

### 2.1 Participants

One hundred and sixty-nine secondary school students (39.3% girls) volunteered to participate during the 2021–2022 academic year in this cross-sectional study. The participants’ characteristics can be seen in Table 1. All the participants performed all the tests (i.e., the original CAMSA, cognitive CAMSA, Corsi and Digit Span tests). The sample was selected from three secondary schools of urban areas in the province of Valencia near Valencia city (Valencia Community, Spain). Those students with cognitive or physical impairments that prevented them from performing the different tests or that could pose a risk to their health were also invited to participate, but were excluded from the statistical analyses.

**Table 1:** Participants’ sociodemographic characteristics.

Sex	Age (years)	Weight (kg)	Height (m)
Girls	13.07 (0.74)	50.1 (9.95)^*^	1.59 (0.06)^*^
Boys	13.3 (1.02)	54.53 (10.9)	1.65 (0.09)
All	13.2 (0.85)	52.5. (10.7)	1.62 (0.08)

^*^indicate significant differences between girls and boys (*p* < 0.05).

The students’ parents or guardians provided signed informed consent forms before the study, and the participants gave their assent orally. The procedures conducted in the study were performed in accordance with the Helsinki Declaration, and the study was approved by the Ethical Committee of the University of XXX (Code 1259844).

### 2.2 Procedures

The researchers first contacted the school principals to explain the study and request their participation. The families were informed about the nature of the project and once they had given their written consent, the measurement protocol was carried out. To complete all the tests, the participants performed two experimental sessions separated by at least 24 h. The CAMSA and Corsi tests were carried out in one session, while the cognitive CAMSA and Digit Span tests were included in the other. The order of the sessions was counterbalanced within the schools to minimize any potential order effects, i.e., whereas the students in one classroom performed the tests in the same order, those in another classroom performed the test in the reverse order (i.e., first the cognitive CAMSA and Digit Span test, followed by the original CAMSA and Corsi tests).

For the completion of both the cognitive and original CAMSA tests, the students were instructed to complete the assessment as quickly as possible while performing the skills to the best of their motor competence. In line with the original procedure, two demonstration trials were provided for each participating class. The first demonstration was performed by a research assistant familiar with the CAMSA test, while explaining each skill thoroughly. The second demonstration was performed by the same research assistant to indicate the effort and speed required to carry out the test. Each student performed two familiarization trials and two formal trials, which were coded *ad hoc*. During the familiarization trials, verbal cues were used only to remind the participants of the next task to be performed, in an attempt to minimize the impact of memory on the task sequence and completion time. These cues consisted of indicating aloud the next task to be performed (e.g., “throw the ball”). The timing began with the command “go” and ended when the participant kicked the soccer ball (Estevan et al., 2023). The time required to perform the original CAMSA test ranged from 14 to 47 s, and the mean completion time being 22.73 ± 5.57 s. During the assessment trials, no feedback was provided on the task performance and no attempt was made to encourage task performance or affect the learner’s performance in any way. In accordance with the protocol (Longmuir et al., 2017), the score of the best attempt was used to calculate the motor competence scores.

The cognitive CAMSA followed the same procedure as the original CAMSA in terms of the students’ performance. In this case, the time taken to perform this test ranged from 15 to 53 s (average 25.36 ± 7.05 s). Both the original and cognitive CAMSA were performed in the school yard. There were some slight modifications in the cognitive CAMSA related to participants’ cognitive performance during motor performance as decision-making events and minor adjustments to the test equipment (i.e., differences in hoop colors, placing throwing targets, and variations in shooting at goal). The cognitive tests were performed in silent classroom using a laptop and open-source software Psychology Experiment Building Language (Mueller and Piper, 2014). The participants received a series of instructions provided automatically by the program for the completion of the tasks, while any doubts were answered by the researcher. The cognitive tasks were conceptualized as measures of executive functions, as in previous studies (Cooper et al., 2016; Pontifex et al., 2019).

### 2.3 Measurements

#### 2.3.1 Cognitive single tests

The Digit Span test, adapted from Wechsler (1949), evaluates working memory and consists of remembering a sequence of numbers presented to the participants and repeating it in the same order (i.e., Digit Span Forward). Since the digital version of the test was used, the numbers were displayed for 1 s on a laptop screen, with a 1-s interval before the next number. The subjects then had to repeat the complete sequence on the laptop. The length of the sequence of numbers starts at three digits and is gradually increased. Two attempts can be made to repeat the numbers. The test is ended when a participant fails both attempts or when they reach the maximum length of 10 numbers. The total number of correct sequences and the longest correctly recalled numerical sequence (i.e., Block Span) were taken as the test outputs. This test has shown moderately high reliability in children aged 6–12 years old (Alloway et al., 2008; Flanagan et al., 2011).

The Corsi test, based on the Digit Span Test (Kessels et al., 2000) was designed to evaluate visuospatial working memory and consists of remembering a sequence of ordered blocks that appear on a computer screen. The procedure steps included (i) nine blocks in pseudorandom positions; (ii) one block is illuminated during 1 s; (iii) a transient period of 1 s between the stimuli; (iv) second block is illuminated; (v) the procedure continues until the number of blocks reach the maximum sequence length; (vi) the subjects indicate the illuminated blocks by clicking on them with the mouse in the correct order. Two familiarization sequences were first performed, in which three blocks were illuminated, after which the test started with a sequence of two blocks. As in the Digit Span test, the participants were allowed two attempts at each sequence. If they failed both attempts or reached the maximum block sequence of nine blocks the test was ended. The scores obtained were the longest correctly recalled sequence (Block Span), the number of correct attempts and the memory span [minimum sequence length (i.e., 2), added to the number of correct attempts, divided by the number of trials in each sequence length (i.e., 2)].

#### 2.3.2 Motor competence

The CAMSA consists of a series of seven movement skills that are completed in a continuous sequence (Menescardi et al., 2022). These skills include three two-footed jumps, sliding along a 3-m distance, returning, catching, and throwing a ball at a wall-mounted target 5 m away, skipping 5 m, performing a one-footed hop in and out of six hoops, and kicking a ball between two cones 5 m apart. The assessment of the children’s performance in the CAMSA involves two key criteria: the time required to complete the circuit (product-based) and the quality of the movement pattern executed or the motor skill score (process-based). The time taken to complete the circuit is recorded and transformed into a point score, known as the time score, ranging from 1 to 14 (Longmuir et al., 2017). Higher values indicate a shorter time taken to complete the circuit. The quality of movement is assessed by 14 skill performance criteria, with one point awarded for each criterion completed correctly, resulting in the skill score (Longmuir et al., 2017). Combining the product and process scores, a total CAMSA score of 28 points is obtained (Longmuir et al., 2017). The best score of two trials is considered for evaluation. The CAMSA has been validated in Spain among students in Primary and Compulsory Secondary Education, as described in Menescardi et al. (2022).

#### 2.3.3 Simultaneous motor competence and cognitive performance

**Figure 1:**
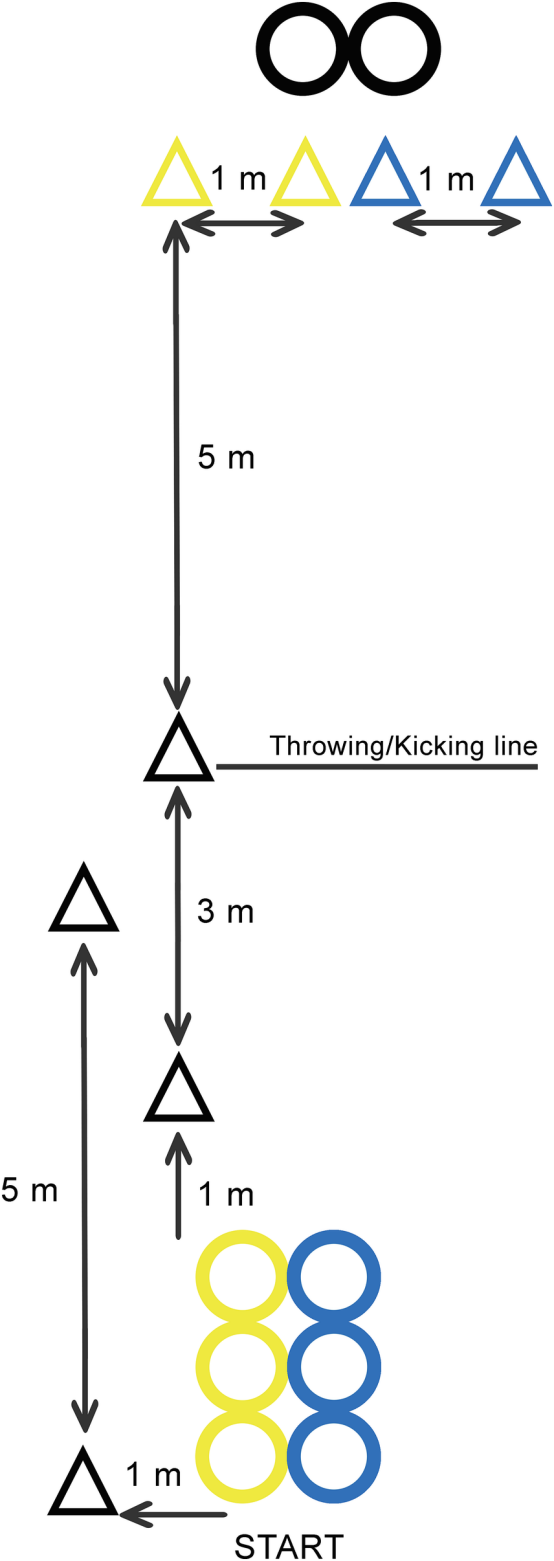
Cognitive CAMSA schedule.

The Cognitive CAMSA was based on the original version of the test with the same motor skills. The changes included were related to the cognitive performance of the participants during the motor performance, with some minor changes to the test equipment including: (i) the three hoops on the left were yellow and the three on the right were blue, (ii) there were two throwing targets in front of the participants with one slightly to the left and the other to the right, and (iii) there were two goal posts, one with yellow cones and the other with blue (Figure 1).

Four decision making events were included in the cognitive assessment: (i) before beginning, the researcher showed a blue or yellow card and the subjects performed the three two-footed jumps inside the hoops of the other color, with 0 or 1 point for a fail or correct performance, respectively; (ii) during the sliding test, the researcher gave some simple numbers to be added before throwing either at the right-hand target if the result was even or at the left-hand target if it was odd, with either a 0 or 1 point for a fail or correct performances respectively; (iii) in skipping, the researcher indicated two numbers to the participants, the first referring to the hoop they should use to start the next motor test (i.e., one-footed jumps) and the second indicated the last hoop in the activity, with 0, 1, or 2 points according to two fails, or to one or two correct performances, respectively; and (iv) after the one-footed jumps, the researcher showed a yellow or blue card and the participants had to kick the ball at the color not shown, with a 0 or 1 point score according to a fail or one or two correct performances, respectively. The cognitive score (from 0 to 5) was the total score, i.e., 0 if no decisions were taken correctly and 5 if all the decisions were correct.

### 2.4 Data analysis

The differences in the skill score, time spent, and total score between the original and cognitive CAMSA were first compared using the Wilcoxon signed rank test. DT interference was then computed (Bustillo-Casero et al., 2017) and represented in boxplots (Oliver et al., 2018) that were influenced by dual tasking.

After determining that not all the adolescents had been similarly affected by dual tasking, the profiles of the DT performance were determined, for which the cognitive score, the skill score, and the time spent in the cognitive CAMSA were included as input variables in a SOM analysis. This analysis was computed on the Matlab R2021a Program (Mathworks Inc., Natick, United States) and the SOM Toolbox (Version 2.0 Beta) for Matlab (Vesanto et al., 1999).

The SOM analysis was used to classify the participants and provide profiles of their similarities in terms of the dependent variables in a three-step procedure (Pellicer-Chenoll et al., 2015), including: (i) the construction of the neuron network (i.e., 11 × 6 neurons map), (ii) the initialization, in which the value or weight of each input variable was assigned to each neuron in two different ways (i.e., randomized and linear initialization), and (iii) a training step to modify the values or weights of the neurons initially assigned by two different training algorithms (i.e., sequential and batch; Oliver et al., 2018).

Several factors influence the modification of the neuronal weights in each iteration during the training. An input vector (i.e., a study case or subject) is entered in the network, after which the neurons in the lattice “compete” to win the input vectors by achieving the smallest Euclidean distance between its weight vector and the input vector, so that the weight vector of the winning neuron has the closest values to the cases in the neuron. All the neurons in the lattice then adapt their weight values closer to the input vector values. The magnitude of the adaptation depends on two processes: (a) the learning ratio, which has a high value at the start of the training process, which is gradually reduced as the training proceeds; (b) the neighbor function, which determines the adaptation of the winning neuron and the rest of the neurons. The adaptation magnitude is negatively associated with the distance between the neuron and the winner. This process is repeated until the training process ends (Pellicer-Chenoll et al., 2015).

Since the final analysis depends on the random procedure (e.g., initialization and entry order of the input vector), the above-described process was repeated 100 times to increase the odds of finding the best solution. 1,600 SOM were obtained in this way because two different training methods, four neighborhood functions and two initialization methods were used (i.e., 100 × 2 × 4 × 2). After multiplying the quantization and topographical errors, the map with the minimum error was then chosen (Pellicer-Chenoll et al., 2015).

After the SOM analysis, a *k*-means method was used to classify the neurons into larger groups, according to the input variables’ characteristics. The number of clusters was set to range between 2 and 10 to avoid an excessive number of profiles. The final number of clusters was the one with lowest Davies-Bouldin index, which were used to describe individual profiles according to the input variables.

The clusters were lastly compared for both the input variables included in the SOM and by gender and single-task performance. The Corsi output variables (i.e., total correct trials, Block Span, and Memory Span), Digit Span (i.e., total number of correct sequences and Memory Span), and original CAMSA (i.e., motor skill score, time to complete the test, and total score) tests were compared between the clusters using the Kruskal-Wallis test with the Dunn test for pairwise comparisons. The Chi-Squared test was used to detect significant associations between cluster membership and gender, with the level of significance set to *p* = 0.05.

## 3 Results

### 3.1 Dual-task interference in the cognitive CAMSA

**Figure 2:**
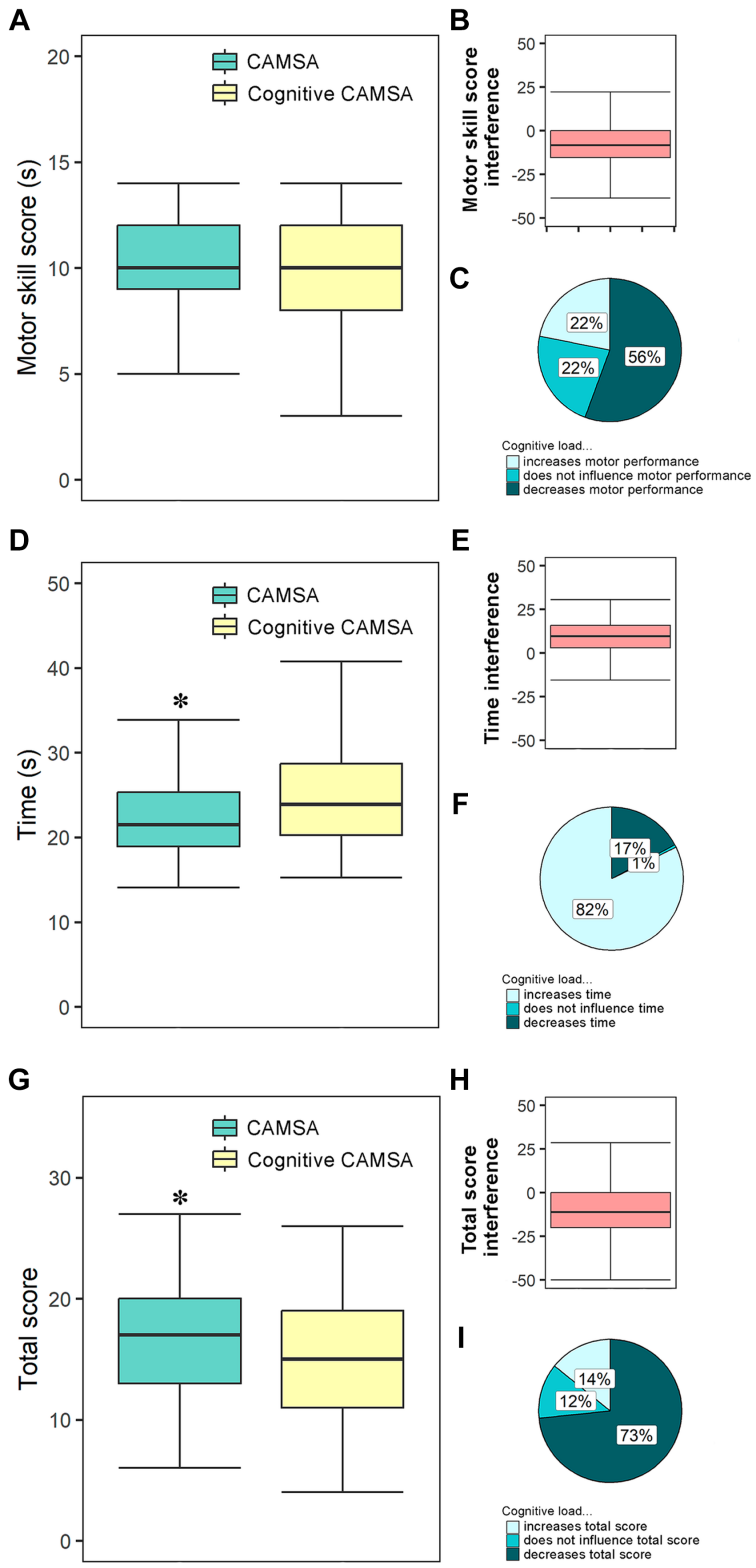
Dual-task interference during the performance of CAMSA test. Layers **(A,D,G)** show box plots of the values obtained in the motor skill score, time, and total score variables both on cognitive and original CAMSA, respectively. Layers **(B,E,H)** show box plots of the interference in the motor skill score, time and total score variables when performing dual tasks (i.e., cognitive CAMSA) compared to when performing single tasks (i.e., original CAMSA) respectively. Layers **(C,F,I)** show the percentage of participants who increase, decrease, or do not change their scores on the variables motor skill score, time, and total score when performing the cognitive CAMSA compared to the original CAMSA, respectively. ^*^indicates significant differences (*p* < 0.05).

All the descriptive data of the variables used in this study are provided in Supplementary material. The results indicate that including cognitive tasks (i.e., decision making) in the cognitive CAMSA significantly reduced the total score while it increased the time spent (Figures 2D,G). Compared to the original CAMSA, the cognitive CAMSA motor skill score therefore did not change significantly (Figures 2A,B). Dual tasking during the cognitive CAMSA thus did not change the students’ motor skill score, whereas the time required to perform the test increased. However, when the DT interference is analyzed (Figures 2E,H), it can be seen that not all the participants had better scores in the original CAMSA (Figure 2I) or increased the time spent in the task (Figure 2F). 14% of the subjects had a higher total score in the cognitive CAMSA than in the original version, whereas 73% had lower total scores and the remaining 12% did not change theirs (Figure 2I). Furthermore, 82% of the subjects required a longer time in the cognitive CAMSA than in the original, while 17% reduced the time spent and the remaining 1% did not require the same time. Although no significant differences were found between the original and cognitive CAMSA in the motor skill scores, in Figure 2C, it can be seen that 22% of the subjects increased their motor skill scores in the cognitive CAMSA, 22% showed no change, while 56% showed a loss of performance (Figures 2B,C,E,H).

### 3.2 Performance profiles of cognitive CAMSA

**Figure 3:**
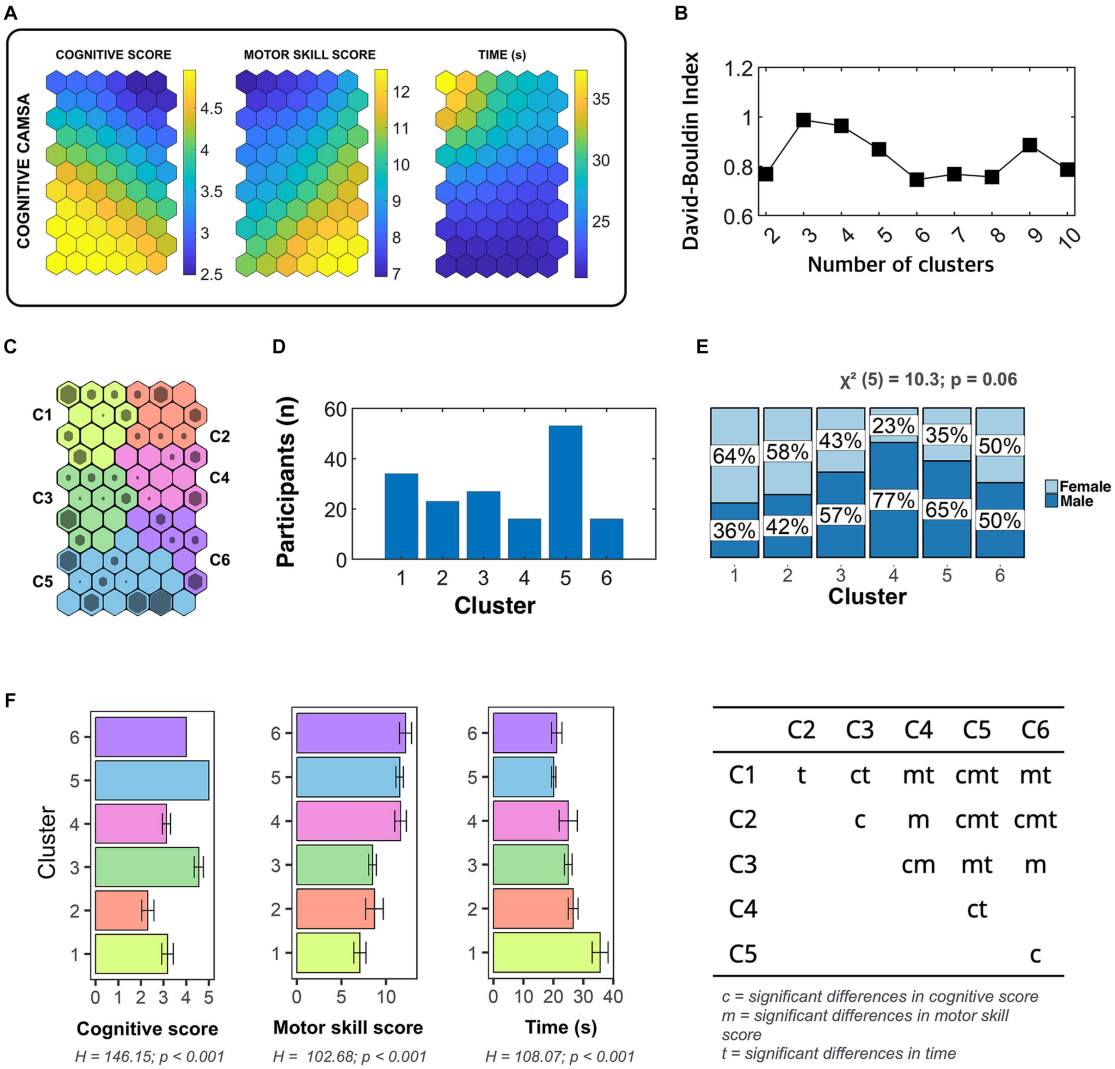
Results derived from Self-Organizing Map analysis in which performance profiles during cognitive CAMSA are described. Panel **(A)** shows the component planes resulted from Self-Organizing Maps analysis on cognitive score, motor skill score, and time spent in complete cognitive CAMSA. Panel **(B)** indicates the Davies-Bouldin index associated with k-means cluster algorithm applied to obtain profiles. Panel **(C)** shows the six clusters obtained using k-means algorithm with the lowest Davies-Bouldin index. Panel **(D)** reports the number of participants allocated in each cluster. Panel **(E)** shows the percentage of girls and boys in each cluster and the chi-square test results. Panel **(F)** represents the mean and standard deviation of each cluster in each of the variables obtained from cognitive CAMSA as well as Kruskal-Wallis test results. Pairwise comparisons are reported in the table of the layer **(F)**, where each letter (i.e., c, m, and t) indicates in which variable significant differences between clusters were obtained.

According to the SOM approach and *k*-means cluster algorithms, using the cognitive CAMSA scores (i.e., cognitive score, skill score, and time spent) as input variables, six performance profiles were obtained (Figure 3). In other words, these six profiles are related to the participants’ performance in a DT situation. The association between cluster and gender was not significant (χ^2^_5_ = 10.3; *p* = 0.06), clusters 4 and 5 having a higher percentage of males, while clusters 1 and 2 were composed of a higher proportion of girls than boys.

A main effect of cluster membership was found on cognitive score (H_5_ = 146.15; *p* < 0.01), motor skill score (H_5_ = 102.68; *p* < 0.001), and time spent (H_5_ = 108.07; *p* < 0.001). Pairwise comparisons revealed that clusters 3 and 5 reported the highest cognitive performance, which, like cluster 2 showed the lowest cognitive performance. Clusters 4, 5, and 6 showed higher values than clusters 1, 2, and 3 in the motor skill score, while the time spent for those in cluster 1 was longer than the rest of the participants in other clusters. The individuals in cluster 5 obtained a higher time spent than those in clusters 2, 3, and 4, while those in cluster 2 required a longer time than those in cluster 6.

### 3.3 Differences between clusters in single task performance

**Figure 4:**
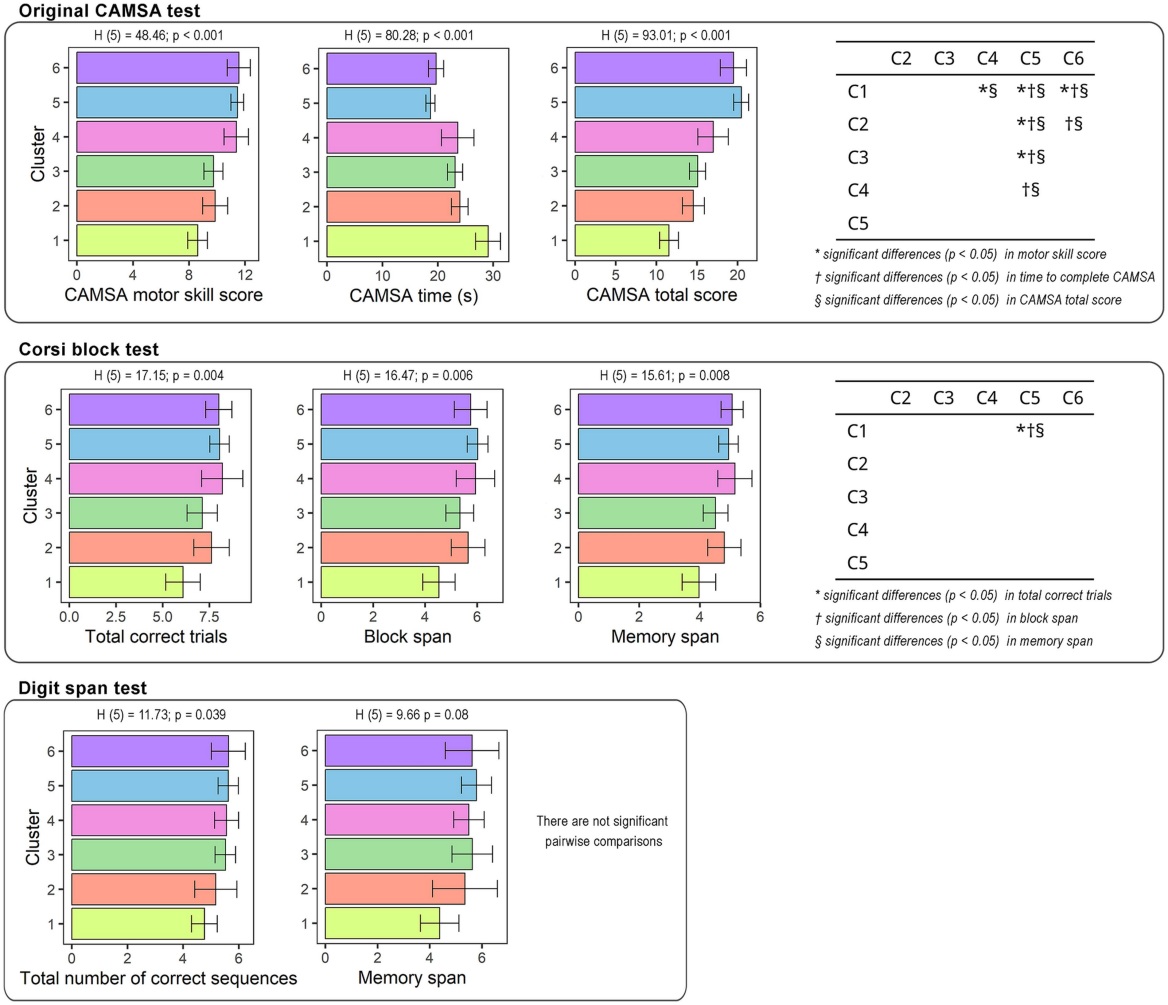
Differences in the variables obtained from original CAMSA, Corsi Block test, and Digit Span test between clusters.

The results revealed a significant effect of cluster membership on the motor skill score (H_5_ = 48.46; *p* < 0.001), time score (H_5_ = 80.28; *p* < 0.001), and total score (H_5_ = 93.01; *p* < 0.001) in the original CAMSA test. Pairwise comparisons (Figure 4) showed that clusters 4, 5, and 6 had higher motor skill scores than cluster 1, while cluster 5 had a higher motor skill score than clusters 2 and 3. In the time score, the participants in clusters 5 and 6 were faster than those in clusters 1 and 2, while clusters 3 and 4 were slower than cluster 5. The total original CAMSA score was also lower in cluster 1 than in 4, 5, and 6, while clusters 5 and 6 showed higher values than 2. The values of those in clusters 3 and 4 were lower than those in cluster 5.

In cognitive performance, there was a main effect of cluster membership on the total correct trials (H_5_ = 17.15; *p* < 0.004), block span (H_5_ = 16.47; *p* < 0.006), and memory span (H_5_ = 15.61; *p* < 0.008) obtained in the Corsi Block test. Pairwise comparisons (Figure 4) revealed that cluster 5 had higher values in total correct trials, block span and memory span than cluster 1. The rest of the clusters showed no significant differences. In Supplementary material, all the pairwise comparisons as well as effect sizes are available.

A main effect of cluster membership was found on the total number of correct sequences (H_5_ = 11.73; *p* = 0.039) in the digit span test, while pairwise comparisons revealed no significant differences between the cluster although accordingly with the *r*-effect size small-to-medium effects were found between cluster 1 and cluster 4, 5, and 6 in both variables (Supplementary material).

## 4 Discussion

Including cognitive demands (e.g., decision making) into field-motor tests could provide a new insight into the motor competence assessment field because, as the setting is maintained, cognitive performance can be assessed in an ecological environment just like active games. The purpose of the current study was thus to design and test a cognitive CAMSA, which includes decision making-based verbal and visual cues in order to assess both motor competence and cognitive performance and to analyze the impact of a cognitive load on motor competence in an ecological setting. In general, and in comparison with the original CAMSA, there were no significant differences in the motor skill score during the application of the cognitive CAMSA. However, the time spent increased, resulting in lower marks in the total score. Interestingly, these results vary widely among the participants in the current study; for instance, some improved their motor skill score (22%), while others did not (22%) or reduced their motor skill score (56%). The same occurred with the time spent, with most the participants increasing the time spent (82%), some reducing it (17%), and a small minority obtaining a similar time (less than 1%). Previous studies have shown an increase in motor performance during DT application, to the detriment of cognitive performance (Schaefer et al., 2008). Other studies have shown how DT motor performance can decline, such as Palluel et al. (2010) and Olivier et al. (2010), who found reduced postural control in adolescents and children, respectively, in a DT compared to simple tasks. The effect of a cognitive load added to a motor task on performance still remains unclear, due to the diversity in the results obtained. However, all these previous results have the defect of considering that DT affects everybody in the same way, the present study being the first to analyze DT interference in a person-centered approach.

From our results it can be concluded that a DT does not affect everyone in the same way. The variables analyzed by the cognitive CAMSA included in the SOM gave rise to six profiles, including several profiles that deserve to be highlighted. The cluster 5 scores excel in both cognitive and motor competence, whether evaluated in isolation or by means of the cognitive CAMSA test, which is considered to be a DT setting. Similarly, cluster 1 shows a lower performance than the rest in both cognitive and motor competence, whether single or dual tasks. Profiles 1 and 5 include the highest number of participants.

It is interesting to note the existence of a profile (Cluster 2) that shows better cognitive performance during seated tests, with a reduced performance in the cognitive CAMSA (i.e., in a DT). In this profile, the individuals exhibited the appropriate levels of motor competence in both the original and cognitive CAMSA. The cognitive CAMSA could be considered as an adequate stimulus to increase the cognitive effort in this type of profile, given the contrasting performances in cognitive CAMSA (DT) and isolated cognitive tasks. On the other hand, it should be noted that a profile (Cluster 3) emerged that stands out for its cognitive performance during the cognitive CAMSA, but not in the simple cognitive tasks. Profile 3 suggests the need to improve motor skills in tasks with a high cognitive load. In terms of cognitive performance, there are groups with heterogeneous levels of cognitive performance in DT and simple tasks. It should be noted that limited significant differences were found between profiles in seated cognitive test. This can be due to a statistical type II error due to a reduced sample size of each cluster. In Supplementary material, some small-to-medium effect size between cluster 1 and clusters 4, 5, and 6 were found in digit span test variables. Future works would check if increasing the sample size of the clusters the differences between them emerge as significant.

While some students (profiles 5 and 6) showed good cognitive performance in the DT and the simple task, others (profile 2) performed worse in the cognitive tasks during DT than in simple tasks, or vice versa (profile 3). Studies by Howell et al. (2016) and Madehkhaksar and Egges (2016) concluded that the complexity of cognitive tasks can influence motor performance during the execution of a DT. On the one hand, Estevan et al. (2018) found reduced performance in motor and cognitive tasks in adolescents as the DT cognitive requirements were increased. On the other hand, Bustillo-Casero et al. (2020) detected better adaptation of postural control in DT as the difficulty level of the cognitive task was raised. So far, no single model has been found to explain DT interference, since it affects individuals differently according to unknown factors, which prevents generalizing theoretical models to the whole population. The cognitive CAMSA seems to emerge as a key tool that enables the precise assessment of student DT performance in order to implement the most suitable learning strategies in schools, as it helps us to accurately identify our students’ characteristics.

As indicated in the previous paragraph, static and moving cognitive assessment are not clearly equivalent, similar to previous research. In the study by Brilliant et al. (2021), discordance between static and moving cognitive test scores was observed. On the other hand, Ramos-Nuñez et al. (2017), confirmed that different executive functions contribute to task performance depending on task difficulty, with modularity being involved in simple tasks and cognitive flexibility in complex tasks. There have been found profiles that perform isolated cognitive tasks correctly, but not during a DT (Cluster 2) and vice versa (Cluster 3), in line with previous studies (Schaefer et al., 2008; Palluel et al., 2010; Howell et al., 2016; Bustillo-Casero et al., 2017). Trying to measure the PL cognitive domain by means of a DT situation (motor competence with a cognitive load) instead of a seated cognitive test may be recommended, since it more closely resembles a real sports situation. In this regard, the cognitive CAMSA allows us to assess a cognitive performance during motor tasks and at the same time make a global motor competence assessment (total score) similar to the one performed in the original CAMSA.

Although there were no significant differences in the men/women ratio in the profiles obtained, this aspect requires attention, since the trend toward significance has been found. As can be seen, some profiles have a higher prevalence of males or females that exhibited clearly distinctive characteristics. The frequency of males in clusters 4 and 5, which have medium to high levels of motor skill scores, both in DT and in isolation (i.e., cognitive CAMSA and original CAMSA) is higher than females, while females appeared associated with cluster 1, with lower levels in the afore-mentioned skills. One reason why motor competence seems to be influenced by gender is that males tend to participate more regularly in physical activity during the early stages (Boraita et al., 2020; Kallio et al., 2020; Abid et al., 2021). This could be attributed to gender-related social conditioning, which could lead to an erroneous physical self-perception and a negative predisposition to sports participation in girls (Cavallo et al., 2015; Corr et al., 2019; Sabiston et al., 2019) and in turn provide women with fewer opportunities to develop motor competence. It thus seems essential to equalize the gender participation in sports activities and to improve female students’ physical self-perception. These findings highlight the need for educational interventions that equalize the results of boys and girls. It is noted that the context conditions females, as in early childhood boys and girls tend to show similar results in motor skills, which can be even higher in the female gender (Rodríguez-Guerrero et al., 2023). Therefore, implementing programs to encourage girls to take up physical activity may be key to achieving this goal (Aguilar Jurado et al., 2018). In addition, the professional training of teachers is a factor that can have a strong influence on the development of motor skills in early childhood for both boys and girls (Honrubia Montesinos et al., 2023).

As has been noted, since different factors can influence performance when carrying out a DT, it seems necessary to examine additional factors which in theory are not considered during its assessment. Those with a more developed PL (frequently those with more sports experiences) are likely to obtain higher motor competence scores (Seidler, 2004; Li et al., 2007; Yang, 2015) and probably also cognitive performance during a motor task. Each individual’s functional perceived difficulty of a task could also be a conditioning factor (Li et al., 2007; Goldhammer et al., 2014; Akizuki and Ohashi, 2015; Aljamal et al., 2019). It is also possible that different people carry out the same task with more or less effort, and this should be taken into account when evaluating a DT. In addition to enjoyment, it is also important to mention the motivation involved in the task. Those who show greater motivation to perform a DT make a greater effort to improve their performance (Ainley, 2006; Li et al., 2007; Shin and Grant, 2019). In future research, it would be interesting to measure all these factors to obtain results closer to DT evaluation in a more realistic way.

Like most, this study is not without its limitations. Despite the fact that previous studies have shown differences by gender in the levels of motor competence (Boraita et al., 2020; Kallio et al., 2020; Abid et al., 2021), no significant association between clusters and gender were found in the present study. This divergence can be due to the fact that we are conducting a person-centered approach while previous studies perform the analysis on a variable-centered approach. Secondly, the lack of a specific scoring scale for the cognitive CAMSA (including relative time score) reduced the total score. Future research should consider the application of a separate scale for cognitive CAMSA scoring in this way. Furthermore, it should be noted that in the cognitive tasks of the cognitive CAMSA there is the possibility that a motor error may occur instead of a cognitive error. For example, it is possible that some participants resolved correctly the question about to what target to throw the ball but technically they fail and hit the incorrect target. This has been categorized as a cognitive error but actually it is a motor skill mistake. It should be noted that of the five cognitive decisions to be made, a motor error is only possible in two (throwing and kicking). Moreover, the authors consider that this error is marginal and does not significantly affect the results obtained. Nevertheless, as an alternative, it would be interesting to ask participants to comment aloud on the side of the throw and kick before performing it. Finally, an important area for attention is the need for extensive validity and reliability testing of the Cognitive CAMSA. The lack of extensive validation and reliability testing may limit the consistency and accuracy of the instrument in providing results across different demographic groups and over time. The recognition of this requirement for further validation and reliability testing underlines the need for future research to ensure a full understanding and robustness of the Cognitive CAMSA as an assessment tool.

Regarding the study’s practical applications, we can highlight on one hand the design of the cognitive CAMSA, which assessed not only motor competence, but also cognitive performance. The cognitive CAMSA could thus be applied in physical education to assess these two PL aspects in secondary school students and also whether it improves over time. We were also able to determine the profiles of adolescents in performing the cognitive CAMSA and to classify individuals into these profiles to determine their general characteristics and any aspects that should be empowered. For example, a student who performs the cognitive CAMSA and is placed in cluster 5 will be an adolescent with high DT performance (both cognitive and motor competence) but will also perform well when executing the tasks separately. In view of these results, educational interventions should be considered in physical education based on improving motor and cognitive performance in DTs.

## 5 Conclusion

In conclusion, a cognitive version of the CAMSA was designed and tested for assessing both motor and cognitive performance, which are elements in the physical and cognitive PL domains. We found that the interference generated by the cognitive CAMSA did not affect all the adolescents equally, i.e., we found six student profiles based on their motor competence and cognitive performance. Some of these profiles belonged to adolescents with a similar performance in both DT and single-task contexts (e.g., cluster 5 and 6). However, others showed high motor competence in single and dual tasking while their cognitive performance declined (e.g., cluster 4) or increased (e.g., cluster 3) in DT compared to single tasks. Adolescents will benefit from the cognitive CAMSA because it allows them to be grouped by their performance and any aspects that require to be fostered.

## Data availability statement

The raw data supporting the conclusions of this article will be made available by the authors, without undue reservation.

## Ethics statement

The studies involving humans were approved by Ethics committee of the University of Valencia. The studies were conducted in accordance with the local legislation and institutional requirements. Written informed consent for participation in this study was provided by the participants’ legal guardians/next of kin.

## Author contributions

SM-G: Data curation, Investigation, Methodology, Visualization, Writing – original draft. IE: Conceptualization, Funding acquisition, Resources, Supervision, Writing – review & editing. JR-M: Data curation, Investigation, Methodology, Writing – review & editing. NO-B: Formal analysis, Investigation, Validation, Writing – review & editing. IV-S: Methodology, Resources, Validation, Writing – review & editing. CM: Conceptualization, Data curation, Project administration, Writing – review & editing. XG-M: Formal analysis, Funding acquisition, Resources, Software, Supervision, Visualization, Writing – review & editing.
